# Screening and application of lactic acid bacteria and yeasts with l‐lactic acid‐producing and antioxidant capacity in traditional fermented rice acid

**DOI:** 10.1002/fsn3.1900

**Published:** 2020-09-24

**Authors:** Na Liu, Song Miao, Likang Qin

**Affiliations:** ^1^ Key laboratory of Plant Resource Conservation and Germplasm Innovation in Mountainous Region (Ministry of Education) Collaborative Innovation Center for Mountain Ecology & Agro‐Bioengineering (CICMEAB) College of Life Sciences/Institute of Agrobioengineering, Guizhou University Guiyang China; ^2^ Teagasc Food Research Centre Cork Ireland; ^3^ School of Liquor and Food Engineering, Guizhou University Guiyang China

**Keywords:** antioxidant capacity, flavor, *K. marxianus* L1‐1, *L. paracasei* H4‐11, L‐lactic acid

## Abstract

In the study, *Lactobacillus paracasei* H4‐11, *Lactobacillus fermentum* D1‐1, *Lactobacillus casei* H1‐8, *Lactobacillus reuteri* H2‐12, and *Kluyveromyces marxianus* L1‐1 were screened from traditional fermented rice acid based on several indicators: L‐lactic acid production capacity (13.46 ~ 19.69 g/kg), antioxidant capacity (DPPH clearance ability of 35.36 ~ 56.89%), and savory flavor indicators. Glutinous rice, quinoa, barley rice, and brown rice were selected to carry out rice acid fermentation. Different viable lactic acid bacteria and yeasts were screened, respectively, in the saccharification, acidification, alcoholization, and late acidification stages. Rice acid fermented with *L. paracasei* H4‐11 and *K. marxianus* L1‐1 in glutinous rice showed the high L‐lactic acid content (23.09 g/kg). The DPPH free radical scavenging ability in rice acid fermented with *L. fermentum* D1‐1 and *L. paracasei* H4‐11, respectively, reached 34.27% and 33.05% in 96 hr. Although quinoa rice acid had the highest L‐lactic acid content (33.74 g/kg) and the DPPH free radical scavenging ability (60.10%), it had the poor taste due to the high astringency intensity and bitter intensity. Rice acid fermented with both *L. paracasei* H4‐11 and *K. marxianus* L1‐1 in glutinous rice showed the highest savory flavor and had the lowest astringency and bitter. *L. paracasei* H4‐11 and *K. marxianus* L1‐1 were the potential strains for the fermentation of rice acid. These results promote the industrial development of Chinese rice acid.

**Practical applications:**

Rice acid as a functional nondairy fermented product is widely accepted by Chinese consumers. However, rice acid fermentation is still a traditional spontaneous process, which causes instability in its flavor and quality. *Lactobacillus paracasei* H4‐11 and *Kluyveromyces marxianus* L1‐1 screened in this study contributed to the improvements in the flavor and antioxidant capacity of rice acid. In addition, glutinous rice was confirmed as the suitable fermentation material of rice acid in the study.

## Graphical abstract

The *Lactobacillus paracasei* H4‐11 and *Kluyveromyces marxianus* L1‐1 were isolated in this study contribute to the improvement of flavor and antioxidant capacity of rice acid. In addition, the mechanism of glutinous rice suitable for growth in rice acid system was verified, making our study particularly relevant. These results would aid in the development of industrial‐scale Chinese acidic fermented rice soup.

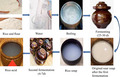

## 1 INTRODUCTION

Sour soup, as one of the traditional foods of Miao and Dong Nationalities, is popular in Guizhou, China. Sour soup mainly includes “white‐acid” (rice acid) prepared with glutinous rice or nonglutinous rice and “red‐acid” prepared with tomato or red pepper as raw materials. Especially, a composite base prepared with two sour soups is used in cooking a large number of foods with different flavors. Rice acid plays an important role in Chinese acidic grain‐fermented soup since it has the antifatigue, anti‐aging, and immunoregulatory functions and can lower cardiovascular disease risks (Yuan, 2010).

In general, rice acid was prepared through the fermentation process involving lactic acid bacteria (LAB) and yeasts. Fermentation is a common food‐processing method that preserves foods and endows them with special flavors (Shen et al., 2018). Previous studies demonstrated that LAB and yeasts had good probiotic properties and produced a variety of compounds such as organic acids, acetaldehyde, methyl alcohols, and esters (Rai et al., 2019; Yadav et al., 2016). A recent study demonstrated that the combination of LAB and *Saccharomyces cerevisiae* produced a flavor profile close to that of the product fermented with *Obushera* (Mukisa et al., 2017). In particular, L‐lactic acid was the most important acid since it could be produced with immobilized *Lactobacillus casei* and agro‐industrial wastes as carbon and nitrogen sources (Thakur et al., 2019). The main nutritional component in foods fermented with LAB is lactic acid. Notably, the human body has only L‐lactic acid dehydrogenase, which can only catalyze L‐lactic acid. The excessive intake of D‐lactic acid or DL‐lactic acid can cause metabolic disorders in the human body and adverse reactions such as acidosis (Narayanan et al., 2004). Therefore, more fermented foods containing more L‐lactic acid should be developed. LAB and yeasts assist in the release and biotransformation of those health‐beneficial compounds via fermentation, increase their bioavailability, and reduce sugar contents in fermented foods (Shen et al., 2018). The fermentation process of rice acid was optimized (Yuan, 2010). However, screening strains of LAB and yeasts from in rice acid has not been reported. The fermentation process with LAB and yeasts is a potential method of improving the quality indicators of rice acid, including acid‐producing capacity and flavor characteristics.

Fermentation with LAB and yeasts is a common process of fermented foods and can improve the potential quality such as antioxidant capacity (Shen et al., 2018; Wang et al., 2014). It is necessary to screen the strains with high antioxidant ability. Menezes et al. (2020) evaluated the phytate hydrolysis and antioxidant activities of some yeast strains and found that 13 strains showed positive results and might improve the nutrient availability of plant‐based foods. Hamidi et al. (2020) indicated that *Rhodotorula mucilaginosa* sp. GUMS16 had the antioxidant and antiproliferative activities. Shen et al. (2018) demonstrated the increases in phenolic, fatty acid, and phytosterol contents and a special antioxidant activity in sweet potato fermented with *Lactobacillus acidophilus*. In the study, common assays and methods were used to screen LAB and yeasts with high antioxidant capacity from fermented rice acid.

However, rice acid fermentation is still a traditional process with a long fermentation period and susceptible to the raw material quality, bacterial contamination, and environmental factors. Therefore, the quality or flavor of rice acid is not stable. Our preliminary investigation showed that rice acid fermentation microorganisms mainly included *Lactobacillus*, yeasts, acetic acid bacteria, and *Leuconostoc* (Jiang et al., 1997).

Therefore, in order to shorten the fermentation period and improve the quality of rice acid, LAB and yeast strains with L‐lactic acid‐producing and antioxidant capacity were screened from traditional fermented rice acid and identified in the study. In addition, this study confirmed that glutinous rice was a suitable raw material for rice acid fermentation.

## 2 MATERIALS AND METHODS

### 2.1 Materials

**FIGURE 1:**
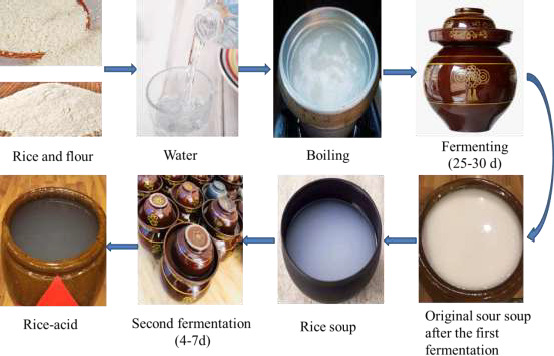
Rice acid processing process

Two types of fermentation samples were selected in the study, including high‐temperature fermentation samples (D1, D1, and D3 from Congjiang, Guizhou, China) and low‐temperature fermentation sample (H1, H2, H3, H4, L1, M1, and M2 from Huangping and Kaili, Guizhou, China). Figure 1 and Table 1 show the process and sample characteristics of different rice acid. The rice acid fermentation process involves two steps. First, a mixture of rice and water is fermented in a fermentation jar at room temperature for 30 days to obtain the “primary rice acid soup.” Second, the primary rice acid soup was subjected to additional fermentation at 28 ~ 35°C for 4 ~ 7 days, thus obtaining in a final rice acid soup product. Samples L1, M1, and M2 came from the enterprise, and the remaining 7 samples were collected from farmers. The samples were produced locally by individuals (farmers) or generated via a standardized process (enterprises) and had different flavors due to different fermentation process. MRS agar medium, potato dextrose agar (PDA) media, MRS liquid medium, and PDB liquid medium were obtained from Bio‐way Biotechnology Co., Ltd. (Shanghai, China). Other chemicals were purchased from Sigma‐Aldrich Co., Ltd., USA.

**TABLE 1:** Samples of rice acid obtained with different fermentation methods

Sample	Location	High temperature/Low temperature	Farmer/Enterprise	Processing process
H1	Huangping	Low temperature (8°C–12°C)	Farmer	Compared to Figure 1, the raw material for the first fermentation is rice and flour.
H2	Huangping	Low temperature (6°C–10°C)	Farmer	Compared to Figure 1, the raw material for the first fermentation is rice and the second fermentation takes 6 d.
H3	Huangping	Low temperature (6°C–10°C)	Farmer	Compared to Figure 1, the second fermentation takes 3 d.
H4	Huangping	Low temperature (8°C–12°C)	Farmer	Compared to Figure 1, the raw material for the first fermentation is rice and the second fermentation takes 6 d.
L1	Kaili	Low temperature (10°C–14°C)	Enterprise	Compared to Figure 1, the raw material for the first fermentation is rice and the second fermentation takes 5 d.
M1	Kaili	Low temperature (13°C–17°C)	Enterprise	Compared to Figure 1, the raw material for the first fermentation is rice and the second fermentation takes 7 d.
M2	Kaili	Low temperature (13°C–17°C)	Enterprise	Compared to the processing of M1, the second fermentation 6 d.
D1	Congjiang	High temperature (60°C–75°C)	Farmer	Compared to Figure 1, the second fermentation takes 7 d and its fermentation near the cooktop indoor.
D2	Congjiang	High temperature (50°C–70°C)	Farmer	Compared to Figure 1, the second fermentation takes 3 d and its fermentation near the cooktop outside.
D3	Congjiang	High temperature (60°C–80°C)	Farmer	Compared to Figure 1, the second fermentation takes 5 d and its fermentation near the stove indoor.

### 2.2 Methods

#### 2.2.1 Screening of LAB strains and yeast strains

##### Isolation and purification of LAB strains and yeast strains

LAB strains were isolated with MRS medium. Modified MRS liquid culture medium was prepared with beef extract (10 g), peptone (10 g), yeast extract (5 g), anhydrous glucose (20 g), Tween‐80 (1 ml), dipotassium phosphate (2 g), sodium acetate (5 g), diammonium hydrogen citrate (2 g), magnesium sulfate (0.58 g), and manganese sulfate (0.25 g) and 1 L of distilled water. CaCO_3_ (10 g) was added to check whether there was a calcium‐soluble circle. After the pH was adjusted to 6.4 ± 0.2, the prepared medium was sterilized at 121°C for 15 min. The modified MRS medium contained 2% agar.

LAB strains were isolated according to the following procedure. The rice acid samples were obtained on the site, sealed in sterile sampling bottles at 4°C, and immediately carried to the laboratory. The samples were immediately diluted by 10^3^, 10^4^, and 10^5^ times with sterile water and spread on modified MRS solid medium, which was incubated at 37°C for 48 hr. The suspected colonies were picked and further purified with streak plate method. The above steps were repeated for 4 to 5 times until a single colony was obtained. The purified single colonies were stored in MRS slant culture medium at 4°C until use. Subcultures were performed every 4 weeks for further analysis.

Yeast strains were isolated according to the following procedure. A sample of rice acid was diluted and spread on PDA medium. The medium was cultivated at 30°C for 3–5 days. Suspected yeast colonies with different colony characteristics were picked and purified with streak plate method.

##### Morphological, physiological, and biochemical characteristics

LAB strains were checked through microscopic observations according to the following procedure. Colonies were picked with needles onto clean slides. After the cells were fixed, gram staining was performed according to the following procedure. After staining with crystal violet and iodine, 95% alcohol and saffron solution was, respectively, added on the slides for decoloring and counterstaining. Then, the slides were placed under the microscope for bacterial observation. The observation results were interpreted based on their staining results. Purple and red, respectively, indicated Gram‐positive and Gram‐negative bacteria. The photograph was captured to record the bacterial morphology. Catalase‐negative and Gram‐positive results indicated LAB strains. Catalase test was performed according to the following procedure. Colonies were picked with an inoculation needle and placed on a clean glass slide. Hydrogen peroxide solution (3%) was added. Catalase‐positive and Catalase‐negative results were determined according to the appearance of air bubbles within 30 s.

Yeast strains were checked through microscopic observations according to the following procedure. Suspected yeasts were transferred into a water‐immersed slide for microscopic observations. The strains with typical yeast characteristics and good gas production performance were cultivated in slant tubes, and then, 30% glycerol was added according to ratio of 1:1 in the tubes, which were preserved in a laboratory refrigerator at −80°C.

#### 2.2.2 Screening of LAB strains and yeast strains with flavor‐producing and antioxidant capacity

##### Acid production assay

The activated strain was inoculated in MRS liquid medium according to 3% inoculation volume and cultured at 37°C for 16 hr, and then, the pH was measured. The total acids were measured according to the method by Cao et al. (2017). The purity of L‐lactic acid was measured according to the method of Moon et al. (2012). The concentration of L‐lactic acid was measured with Amplite™ Colorimetric L‐Lactate Assay Kit (AAT Bioquest Inc. USA).

##### Survival at pH 2.0 and pH 3.0

According to the method described by Ramos et al. (2013), activated strains were, respectively, inoculated in MRS liquid medium with different pH values (pH 2.0 and pH 3.0) according to the inoculation volume of 3% and cultivated at 37°C for 16 hr, and then, OD_600 nm_ was measured.

##### Bile salt tolerance assay

Activated strains were inoculated according to the inoculation volume of 3% in MRS liquid media containing different concentrations of bile salts (0.3 g/L and 0.6 g/L) and cultivated at 37°C for 16 hr, and the OD_600 nm_ was measured.

##### Antioxidant property assay

###### Preparation of the sterile saline suspension of LAB and yeasts

Single colonies of LAB and yeasts with good growth conditions obtained through the above separation steps were respectively inoculated into 3 ml of MRS and PDB liquid medium and respectively cultivated at 37°C for 18–20 hr and 30°C for 48–72 hr. The culture medium was inoculated in 50 ml of MRS and PDB liquid medium according to the inoculation volume of 2% and allowed to stand for 18 hr to obtain the culture medium of the strains. The cells were collected by centrifugation at 1,000 rpm for 6 min at 4°C. After washing twice with sterile physiological saline, the cells were resuspended and the cell density was adjusted to 1.01 ± 0.05 × 10^8^ CFU/ml.

###### Determination of the ability to scavenge 1,1‐diphenyl‐2‐trinitrophenylhydrazine (DPPH)

LAB strains and yeast strains were tested to explore their ability to scavenge free radicals (DPPH) according to the following steps. First, 2 ml of saline suspension of lactic acid bacteria to be tested was added to 2 ml of 0.4 mmol/L DPPH solution, mixed well, placed in the light‐shielding environment at room temperature for 30‐min reaction, and then centrifuged at 8,000*g* for 10 min. Then, the OD_517 nm_ of the supernatant was measured. In the blank group, DPPH was replaced by an equal volume of absolute ethanol. The control group samples were replaced by an equal volume of distilled water. A mixture of the equal volume of distilled water and absolute ethanol was used as the calibration sample to adjust the OD_517 nm_ to zero. The experiments were performed in triplicate.

The scavenging rate is calculated according as:

$$\text{Scavenging}\;\text{rate}\;\text{of}\;\text{DPPH}=\left(A_{\text{control}}‐A_{\text{sample}}\right)/A_{\text{control}}\times 100\%,$$

where *A*_control_ is the absorbance of the control group, and *A*_sample_ is the absorbance of the sample group.

###### Ferric reducing antioxidant capacity (FRAP reducing ability)

First, 150 μl of sample solution was mixed with 2,850 μl of FRAP solution (300 mmol/L sodium acetate buffer solution (pH = 3.6): 20 mmol/L FeCl_3_: 10 mmol/L TPTZ = 10:1: 1; V/V/V). The reaction proceeded at 37°C for 10 min, and OD_593 nm_ was measured by using a microplate reader. With distilled water as a blank instead of the sample and 0, 100, 200, 300, 400, 500, and 600 μmol/L Trolox as standard solutions, the standard curve was plotted according to the above steps. The results are expressed in micromoles of Trolox equivalent (TE) per gram dry basis (μmol TE/g DW).

###### Determination of hydroxyl radical scavenging ability (OH^–^)

According to the reported method with minor modifications (Mu et al., 2018), hydroxyl radical scavenging ability was determined. The reaction mixture contained 1.0 ml of PBS (0.02 mol/L, pH = 7.4), 0.5 ml of o‐phenanthroline (2.5 mmol/L), 0.5 ml of FeSO_4_ (2.5 mmol/L), 0.5 ml of H_2_O_2_ (2.0 mmol/L), and 0.5 ml of bacterial suspension. After reaction for 1 hr in water bath at 37°C, the reaction mixture was centrifuged at 4,000 *g* for 10 min and OD_536 nm_ of the supernatant was measured as A_sample_. OD_536 nm_ of the blank in which the sample was replaced with distilled water was measured as A_blank_. Distilled water was used to replace H_2_O_2_ as the control group to measure OD_536 nm_, which was recorded as A_control_.

##### Screening of aroma‐producing yeasts

After the isolation and purification of the yeast strains, they were respectively cultured in PDB liquid medium in a shaker (30°C, 120 r/min) for 1 d. The strains capable of producing mellow aroma and ester aroma were screened. The aroma profiles were investigated by sniffing and sensory evaluation, and the keyway was to smell the aroma according to the previous method (Wang, 2016).

#### 2.2.3 Strain identification with molecular biological tools

##### Bacterial and fungal DNA extraction

Bacterial and fungal DNA extraction kits were, respectively, used for DNA extraction according to the kit instructions of BayGene/Sigma‐Aldrich.

##### PCR amplification and sequencing

After the extraction of bacterial DNA, the genotypic identification of selected strains was performed by PCR. PCR reaction system (25 μl in total) was composed of Taq PCR Master Mix (12.5 μl), DNA template (2 μl), Primer 27 *F* (10 μmol/L) (1 μl), Primer 1492 R (10 μmol/L) (1 μl), and sterilized ddH_2_O (8.5 μl). PCR amplification program was set as follows: predenaturation at 94°C for 5 min, 35 cycles of denaturation at 94°C for 1 min, annealing at 55°C for 1 min, and extension at 72°C for 2 min, followed by the extension at 72°C for 10 min. The products were detected by 1% agarose gel electrophoresis and sequenced with universal primers 27 *F* (5ʹ‐AGAGTTTGATCCTGGCTCAG‐3ʹ) and 1492 R (5ʹAAGGAGGTGATCCAGCCGCA‐3ʹ). 16S rRNA sequence was completed by Thermo Fisher Scientific Inc.

After extracting the fungal DNA, PCR was performed with the primers of NL1 (5ʹ‐GCA TAT CAATAAGCG GAG GAA AAG‐3ʹ) and NL4 (5ʹ‐GGTCCG TGTTTC AAG ACG G‐3ʹ). PCR reaction system (25 μl) was composed of 2 μl of DNA template, 1 μl of NL1, 1 μl of NL4 (10 μmol/L) each, 12.5 μl of 2 × Taq PCR MasterMix, and 8.5 μl of ddH_2_O. PCR amplification program was set as follows: predenaturation at 95°C for 5 min, 40 cycles of denaturation at 94°C for 40 s, annealing at 55°C for 40 s, and extension at 72°C for 30 s, followed by the extension at 72°C for 10 min. PCR products were detected by 1% agarose gel electrophoresis and sequenced with the primers (NL1 and NL4). Fungal ITS sequence was completed by Thermo Fisher Scientific Inc.

##### Construction of phylogenetic tree

After sequencing PCR products, the obtained gene sequences were checked with Chromas 2.33 software and compared with the known sequences in GenBank by BLAST software for strain identification. The sequences were aligned with the corresponding model strains and outgroup strains in Dnaman 5.0 software, and the UPGMA method was used for 1000 bootstrap tests to construct a phylogenetic tree (Suzuki et al., 2002).

#### 2.2.4 Preparation methods of rice acid with different cereals raw materials

Rice acid was fermented according to the following method. First, different cereals (glutinous rice, quinoa, barley rice, and brown rice) were broken with a high‐speed pulverizer and sieved twice with an 80‐mesh sieve to prepare rice flour. Then, water was added into rice flour according to the proportion of 1.5% rice flour: 98.5% water and boiled under stirring conditions to obtain rice soup. Then, boiled rice soup was gelatinized for 30 min in a water bath under stirring conditions to prevent uneven gelatinization and local deterioration of the gelatinization solution. Gelatinization time was 30 min and the temperature at 60°C. Then, 1.0% α‐amylase was added into gelatinized rice soup, which was liquefied for 30 min and then sterilized at 95°C for 20 min. After the rice soup was cooled to about 30°C, *Kluyveromyces marxianus* L1‐1 was inoculated into rice soup according to the proportion of 0.1% and strains of *L. paracasei* H4‐11 and, *Lactobacillus fermentum* D1‐1 were be inoculated according to the proportion of 8.0% into rice soup of four cereals. Then, the mixtures were, respectively, added in a fermentation jar and fermented in a constant temperature incubator at 40°C for 4 d.

#### 2.2.5 Sensory evaluation and electronic tongue measurement

Quantitative descriptive sensory analysis method was used to evaluate the rice acid samples according to a ten‐point interval scale (0 = none, 9 = extremely strong). The sensory evaluation panel was composed of 15 females and 5 males (20–30 years old). Sensory sessions took place in a sensory laboratory, which met international standards for a test room. According to the discussion and pre‐experiment results with the panel, the sensory evaluation was performed in coded tasting cups containing 100 ml of water with 1.5 g of samples. Samples were presented in a random sequence (Lin et al., 2018). Meanwhile, the aroma profiles were investigated by sniffing (Wang, 2016). In addition, 8 ml of rice acid filtrate was diluted to the volume of 80 ml for measurements with an electronic tongue (Insent SA‐402B, Atsugi‐chi, Japan). The response intensity of each sensor was measured with an Ag/AgCl reference electrode, which was the most commonly used in this field. Each sample was measured after the electric potentials of all membranes had been stabilized in standard solutions. The detailed operation process was performed according to the method of Phat et al. (2016).

### 2.3 Statistical analysis

All experiments were performed in triplicate. The data are expressed as mean ± *SD*. The mean and standard deviation (*SD*) were calculated, and other statistical analyses were carried out in Microsoft R Excel 2010 software package and SPSS 20.0.

## 3 RESULTS AND DISCUSSION

### 3.1 Morphological analysis of different LAB strains and yeast strains

**FIGURE 2:**
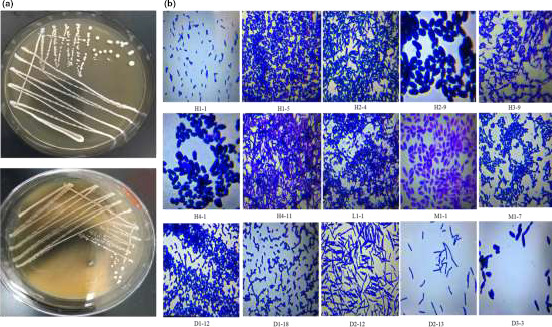
(a) The morphology of lactic acid bacteria colonies. (b) The microstructures of 15 different lactic acid bacteria under the microscope

**FIGURE 3:**
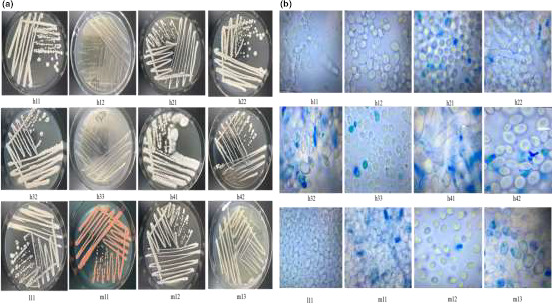
(a) The morphology of 12 different yeasts strains colonies. (b) The microstructures of 12 different yeasts strains under the microscope

Different strains showed different colony states and microstructures under the microscope in this study. LAB colonies showed similar shapes (Figure 2a), and the colony size was in the range of 0.5–2.0 mm. The 86 LAB strains showed different microstructures under the microscope. Figure 2b shows 15 LAB strains. Most of them were rod‐shaped (long rod, medium rod, and short rod). The minority of them were oval, and only a few bacteria were spherical. Figure 3a shows the diverse colony morphology, and red, yellowish, and white colonies were observed. The colonies of yeast strains were spherical or wrinkled. The microstructures of yeast strains under the microscope showed significant differences. Figure 3b shows 12 yeast strains with the shapes of spheroids or ellipsoids.

### 3.2 Acid‐producing capacity of different LAB and yeasts

In the study, 86 potential LAB strains were selected for the analysis of physiological and biochemical characteristics (the data were not shown). Sixty‐seven strains were Gram‐positive and catalase‐negative strains. Nineteen strains were Gram‐negative and catalase‐positive strains, indicating that 19 strains were not lactic acid bacteria. Based on the calcium‐soluble circle, 67 strains of LAB were screened in terms of pH and acid production (Table 2). The acid content in different strains was 5.57–19.69 g/kg. The strain H4‐11 had the highest acid content (19.69 g/kg), and the strains of H2‐1, H1‐8, H1‐6, H1‐3, H3‐9, H3‐5, and H3‐10 had the relatively high acid content. Consistently, in the previous study (Moon et al., 2012), *Lactobacillus paracasei subcasei* CHB2121 produced 192 g/L‐lactic acid in a medium containing 200 g/L glucose. The trends of pH value of LAB showed the significant differences. The pH range of different strains was 3.85–5.92. The pH and total acid content were significantly different among different strains (*p* < .05). Thirty‐three yeast strains had a weaker acid production capacity than LAB strains. The acid contents of yeast strains were 0.92–11.33 g/kg. The acid contents of the strains h1‐1, h1‐2, h2‐2, h3‐2, h3‐3, l1‐1, l1‐3, m1‐2, m2‐1, and m2‐2 were higher than 6.00 g/kg, indicating that the acid production capacity of LAB was significantly different from that of yeast strains (*p* < .05). Consistently, in the previous study (Domizio et al., 2016), LAB strains showed the higher production of lactic acid than yeast strains in fermented beer.

**TABLE 2:** Acid‐producing capacity of lactic acid bacteria and yeasts

Stain	pH	Total acids (g/kg)	Stain	pH	Total acids (g/kg)	Stain	pH	Total acids (g/kg)
Lactic acid bacteria								
H1‐1	3.98	14.63	H3‐4	4.77	8.67	M1‐3	4.05	9.21
H1‐2	4.14	12.66	H3‐5	4.36	15.66	M1‐8	4.77	7.99
H1‐3	4.56	17.22	H3‐6	3.98	12.72	M1‐10	4.93	8.44
H1‐4	4.69	9.36	H3‐7	4.15	11.59	M1‐11	5.87	7.23
H1‐5	3.97	13.23	H3‐8	4.85	8.06	M1‐12	5.12	7.62
H1‐6	3.91	18.23	H3‐9	4.07	15.87	D1‐1	3.87	14.67
H1‐7	4.61	9.23	H3‐10	4.03	15.42	D1‐5	3.99	14.53
H1‐8	3.89	18.68	H4‐1	3.97	12.79	D1‐7	3.97	14.12
H1‐9	3.86	13.84	H4‐4	4.49	11.59	D1‐11	3.87	14.19
H1‐10	3.91	15.75	H4‐7	4.72	9.45	D1‐12	3.89	14.63
H1‐11	4.42	6.53	H4‐9	5.81	6.98	D1‐14	3.96	14.15
H1‐12	4.84	6.75	H4‐10	5.73	7.14	D1‐16	3.87	14.29
H2‐1	3.97	18.57	H4‐11	3.85	19.69	D1‐17	3.97	14.03
H2‐2	4.24	13.91	H4‐12	4.24	13.46	D1‐18	3.89	14.61
H2‐3	4.49	8.15	L1‐1	4.38	7.69	D2‐1	3.92	14.17
H2‐4	4.25	13.17	L1‐2	5.96	6.64	D2‐2	3.88	14.59
H2‐5	4.24	13.23	L1‐4	5.91	6.69	D2‐10	3.94	14.27
H2‐6	4.25	11.55	L1‐5	5.87	6.26	D2‐11	3.89	14.39
H2‐10	4.48	13.17	L1‐6	5.24	5.52	D2‐12	3.92	14.22
H2‐13	4.46	11.59	L1‐7	5.91	5.26	D3‐1	3.95	14.54
H3‐1	4.28	13.165	L1‐8	4.36	13.615	D3‐2	3.93	14.13
H3‐2	4.13	12.6	L1‐11	5.92	6.435			
H3‐3	4.4	12.33	M1‐1	4.51	12.375			
Yeasts								
h1‐1	3.92	11.73	l1‐2	5.98	5.18	d1‐5	4.52	3.94
h1‐2	3.92	9.11	l1‐3	3.91	11.33	d1‐6	4.55	4.73
h2‐1	4.49	3.91	l1‐4	6.18	4.73	d1‐7	4.49	4.5
h2‐2	4.06	11.03	m1‐1	4.55	4.28	d2‐1	4.84	1.69
h2‐3	4.55	4.61	m1‐2	4.5	8.33	d2‐2	4.91	2.14
h3‐1	4.79	2.36	m2‐1	4.05	11.03	d2‐3	4.91	2.36
h3‐2	3.98	8.78	m2‐2	4.09	10.64	d3‐1	4.88	0.92
h3‐3	4.54	6.53	d1‐1	4.54	4.05	d3‐2	5.09	5.74
h4‐1	4.51	5.96	d1‐2	4. 45	4.05	d3‐3	4.78	3.04
h4‐2	4.56	4.73	d1‐3	4.53	4.39	d3‐4	4.74	2.36
l1‐1	5.98	6.96	d1‐4	4.49	5.51	d3‐5	6.21	5.63

### 3.3 Analysis of the aroma‐producing capacity of yeast strains

Different yeast strains produced different flavor characteristics (Table 3). The volatile flavors produced by yeast strains screened from rice acid were alcoholic flavor, ester flavor, fruit sweet flavor, and acidity formed in the acid production process. In terms of the aroma‐producing performance, 30 yeast strains were selected for the comparative analysis through the sensory evaluation and sniffing method (Wang, 2016). Finally, 6 yeast strains with good aroma‐producing capacity (h22, h31, h32, h33, l11, and m21) were selected for antioxidant experiments. In the future study, we will focus on volatile flavor components such as ethyl acetate and acetic acid, which promote the flavor accumulation of rice acid inoculated with yeast strains.

**TABLE 3:** Analysis of the aroma‐producing capacity of different yeasts

Strain	Aroma description	Strain	Aroma description
h11	Yeast‐specific flavor	l11	Mellow and slightly sweet
h12	Fragrance	l12	Yeast‐specific flavor
h13	STRONG sour taste	l13	Slight ester fragrance
h21	Sour	l17	Floral and mellow
h22	Strong mellow fragrance	m11	Alcoholic and slightly eggy
h23	Ester and a certain sweet	m12	Light scent
h24	Eggy and mellow	m13	Yeast‐specific flavor
h212	Rich sweet wine flavor	m14	Sour and mellow
h25	Yeast‐specific flavor	m15	Slightly eggy fragrance
h31	Sweet and fragrance	m21	Rich ester fragrance
h32	Light and mellow	m22	yeast‐specific flavor
h33	Strong mellow Fragrance	m23	Yeast‐specific flavor
h41	Fragrance	m24	Sour and ester
h42	Less sour	d1‐2	Stronger yeast flavor
h43	Yeast‐specific flavor	d2‐1	Stronger yeast flavor

### 3.4 Survival test at pH 2.0 and 3.0 and bile salt tolerance assay

Based on the acid‐producing properties of LAB, 37 LAB strains with large acid production and low pH were selected for acid and bile salt resistance experiments (Table 4). When pH decreased from 3.0 to 2.0, the growth of LAB was inhibited to some degree. Most of the LAB strains had the suitable acid resistance at pH = 3.0, and OD_600 nm_ range was 0.11–1.59. The OD_600 nm_ values of strains M1‐12, D2‐12, and D3‐2 were, respectively, 0.037, 0.02, and 0.05, indicating that the three strains had the poor acid tolerance. The overall performance of these strains at pH = 2.0 was slightly inferior, indicating that the screened LAB strains showed inconsistent activities in different acid environments. The data of bile salt tolerance experiments showed that the LAB activity was significantly inhibited under 0.3 to 0.6 g/L bile salt. Strain D1‐18 showed the best bile salt tolerance, whereas strain M1‐6 had the worst bile salt tolerance. Except M1‐6, all strains grew well under 0.3 g/L bile salt. The previous study (Kaktcham et al., 2018) demonstrated that the bile salt tolerance was a fundamental property required for probiotic LAB to survive. In our study, we screened the potential functional LAB strains for rice acid fermentation.

**TABLE 4:** Determination of acid at pH 2.0 and 3.0 and bile salt at 0.3 g/L and 0.6 g/L tolerance of lactic acid bacteria

Strain	pH = 3.0	pH = 2.0	0.3g/L bile salt	0.6g/L bile salt	Strain	pH = 3.0	pH = 2.0	0.3g/L bile salt	0.6g/L bile salt
H1‐1	0.27	0.20	1.80	0.84	M1‐8	0.11	0.08	0.86	0.40
H1‐2	0.16	0.16	1.55	0.38	M1‐12	0.04	0.03	1.01	0.03
H1‐5	0.29	0.19	2.02	0.60	D1‐1	1.43	0.17	2.01	0.96
H1‐6	0.28	0.19	2.36	0.77	D1‐5	1.44	0.22	1.67	0.73
H1‐8	0.22	0.17	1.82	0.46	D1‐7	1.40	0.18	1.90	0.80
H2‐4	0.24	0.20	1.81	0.97	D1‐11	1.36	0.20	1.84	0.76
H2‐6	0.13	0.12	1.57	0.41	D1‐12	1.41	0.18	1.95	0.83
H2‐10	0.17	0.16	1.73	0.71	D1‐14	1.35	0.18	1.83	0.78
H2‐13	0.32	0.15	1. 18	0.84	D1‐16	1.59	0.22	2.06	1.11
H3‐2	0.24	0.17	1.74	1.12	D1‐17	1.19	0.21	1.68	0.58
H3‐3	0.59	0.13	1.85	1.02	D1‐18	0.67	0.19	2.11	1.24
H3‐4	0.24	0.20	1.84	1.09	D2‐1	1.39	0.24	1.87	0.75
H3‐7	0.11	0.10	1.22	0.33	D2‐2	1.34	0.18	1.86	0.75
H3‐9	0.14	0.13	1.14	0.22	D2‐10	1.45	0.30	1.83	0.56
H4‐1	0.51	0.18	1.86	0.27	D2‐11	1.37	0.17	1.91	0.81
H4‐11	0.21	0.17	1.92	1.04	D2‐12	0.02	0.01	1.88	0.75
H4‐12	0.28	0.19	1.99	0.61	D3‐1	1.32	0.24	1.84	0.75
L1‐8	0.14	0.13	1.43	0.49	D3‐2	0.05	0.04	1.01	0.14
M1‐2	0.16	0.13	1.44	1.01					

### 3.5 Antioxidant capacity

The 16 LAB strains and 6 yeast strains were selected for antioxidant capacity tests with DPPH, FRAP, and hydroxyl radical (Table 5). The DPPH scavenging rate of LAB strains was 34.40%–63.79%, and the total FRAP reducing ability of LAB strains was 55.91–662.61 μmol TE/g DW. The hydroxyl radical scavenging ability of LAB strains was 47.23%–50.99%. The DPPH scavenging rate of yeast strains was 32.91%–52.47%, and the total FRAP reducing ability of yeast strains was 82.17–540.71 μmol TE/g DW. The hydroxyl radical scavenging ability of yeast strains was 45.96%–48.95%. These strains showed different antioxidant capacities. In particular, LAB strains H4‐11 and D1‐1 had unique antioxidant activity. Among various yeast strains, h33 had the highest DPPH scavenging ability and l11 had the highest value of FRAP. Compared with LAB strains in the previous study (Czyżowska et al., 2017), some strains in our study had the higher antioxidant activity including DPPH scavenging ability and FRAP reducing ability and will be applied in rice acid fermentation in the future study.

**TABLE 5:** Antioxidant capacity included scavenging ability of DPPH, FRAP, and OH‐ of lactic acid bacteria and yeasts

Strain	DPPH scavenging ability (%)	FRAP reducing ability (μmolTE/g DW)	The hydroxyl radical OH‐scavenging ability (%)	Strain	DPPH scavenging ability (%)	FRAP reducing ability (μmoTE/g DW)	The hydroxyl radical OH‐scavenging ability (%)
Lactic acid bacteria							
H1‐1	39.48	55.91	49.71	M1‐2	38.79	68.91	47.70
H1‐5	38.86	504.76	47.74	M1‐8	34.40	157.07	48.14
H1‐8	39.49	584.34	50.99	D1‐1	56.89	585.38	49.69
H2‐4	46.00	577.06	50.51	D1‐13	47.84	645.71	48.44
H2‐13	49.03	662.61	50.75	D1‐14	56.47	439.75	50.25
H3‐2	39.84	494.62	51.49	D1‐16	63.79	491.50	47.23
H4‐11	49.71	380.45	50.12	D1‐18	51.85	278.25	50.29
H4‐12	34.61	227.54	49.34	D2‐11	52.21	306.34	50.95
Yeasts							
h22	35.30	530.77	47.89	h33	52.47	207.80	48.95
h31	36.33	105.06	48.91	l11	35.39	540.71	48.12
h32	35.72	261.09	45.96	m21	32.91	82.17	48.18

### 3.6 Growth curves of different LAB strains and yeast strains

**FIGURE 4:**
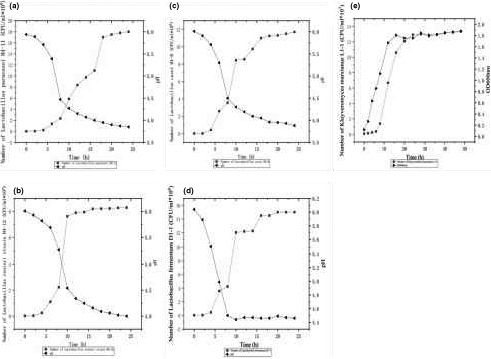
The growth curve was plotted with the colony count and pH of (a) *Lactobacillus paracasei* H4‐11, (b) *Lactobacillus reuteri* H2‐12, (c) *Lactobacillus casei* H1‐8, and (d) *Lactobacillus fermentum* D1‐1. (e) The growth curve was plotted with the colony count and the absorbance at OD600 nm of *K. marxianus* L1‐1

The high‐quality LAB strains H4‐11, H4‐12, M1‐8, and D1‐1 were selected in terms of acid production performance and antioxidant indicators. The growth curves were plotted with the colony count and pH of the 4 LAB strains (Figure 4a–d). In fact, a minimum level of 10^6^ CFU of viable probiotic bacteria per milliliter or gram of foods is accepted. The growth trend of LAB strain H4‐11 was consistent with that of Gallo et al. (2018). In a similar report, *Lactobacillus helveticus* KLDS1.9204 had the highest acid production among 27 LAB strains (Ai et al., 2015). Based on comprehensive acid production, alcoholic and ester‐producing properties, and antioxidant capacity, a high‐quality yeast L1‐1 strain was selected as OD_600 nm_ in the growth curves (Figure 4e) indicated that yeast strain L1‐1 had the better growth ability than other yeast strains.

### 3.7 Identification of different LAB strains and yeast strains with molecular biological tools

**FIGURE 5:**
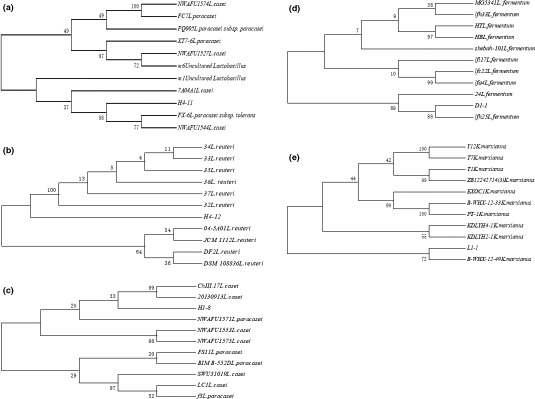
Phylogenetic tree of (a) *L. paracasei* H4‐11, (b) *L. fermentum* D1‐1, (c) *L. casei* H1‐8, (d) *L. reuteri* H2‐12, and (e) *K. marxianus* L1‐1, based on the homology of a 16S rDNA sequence. The numbers at each branch point indicate the percentage supported by bootstrapping with 1,500 repetitions

The high acid production in Chinese traditional fermented rice acid is ascribed to the strains with the high acid‐producing capacity. Due to the interactions among different strains, the unique taste and aroma are formed in fermented foods (Qin & Ding, 2007). A phylogenetic tree was constructed based on 16S rRNA and ITS rRNA sequences of strains (Figure 5). The identified strains included *Lactobacillus casei*, *L. paracasei*, *Lactobacillus reuteri*, *L. fermentum*, *Lactobacillus harbini*, *Lactobacillus vienii*, *Lactobacillus brucei*, *Lactobacillus parasiticum*, *Enterococcus faecalis*, and *Lactobacillus lactis*, *L. casei*. Herein, *L. paracasei*, and *L. fermentum* were the dominant LAB strains. This result indicated that many strains in our study were probiotics, which played a significant role in human health (Kerry et al., 2018). Although some strains were reported, the strains screened by different systems had different physiological and biochemical characteristics, such as *L. paracasei* H4‐11 in this study. When the glucose content in the medium was not high, the acid content was 19.69 g/kg in our study. Consistently, in the previous study (Hassan & Bullerman, 2008), *L. paracasei* isolated from sourdough bread had the unique acid‐producing capacity and antifungal activity.

Based on the consideration of all the indicators in this study, four LAB strains (H4‐11, H1‐8, D1‐1, and H4‐12) with the good characteristics were finally selected to establish a phylogenetic tree. Strain H4‐11 was *L. paracasei* whose sequence homology with *L. paracasei FX‐6* reached 99.79%. Strain H1‐8 was *L. casei* whose sequence homology with *L. casei* 20130913 reached 99.58%. The sequence homology between *L. fermentum* D1‐1 and *L. fermentum* lfh25 was 98.50%, and the sequence homology between *L. reuteri* H4‐12 and *L. reuteri* JCM1112 was 92.18%. H4‐12 might be a new strain, but it requires to be verified in subsequent experiments. Based on the results of fungal ITS sequences, the identified yeast strains included *K. marxianus*, *P. fermentans*, *Rhodotorula spp*., *Candida*, *P*. *pastoris*, *P. mansurica*, and *C*. *albicans*. L1‐1 selected for the construction of a phylogenetic tree was an ester‐producing and alcohol‐producing strain. Strain L1‐1 was determined as *K. marxianus* since it had 100% homology with the strain *K. marxianus* B‐WHX‐12‐33.

### 3.8 Study on the performances of LAB strains and yeast strains in the fermentation of rice acid

#### 3.8.1 Strain characteristics in rice acid fermented with different raw materials

**FIGURE 6:**
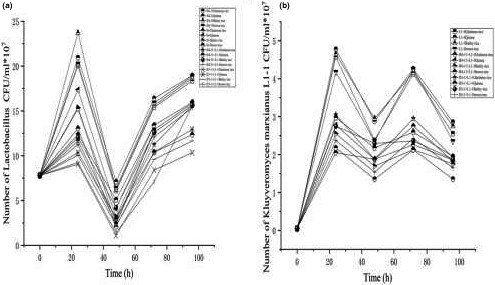
Dynamic changes in the quantity of (a) *Lactobacillus* and (b) *K. marxianus* of *L. paracasei* H4‐11 and *K. marxianus* L1‐1 fermented rice acid compared with *L. fermentum* D1‐1 and *K. marxianus* L1‐1 fermented rice acid by different raw materials included glutinous rice, quinoa, barley rice, and brown rice

The traditional fermentation way of rice acid was simulated. After four days of fermentation, the quantities of LAB and yeast in rice acid fermented with different strains in different raw materials showed significant differences (*p* < .01, Figure 6). The quantity of *L. paracasei* H4‐11 peaked after 24‐hr fermentation and reached 2.37 × 10^9^ CFU/ml in barley rice acid. In rice acid fermented with the other three cereals (glutinous rice, quinoa and brown rice), the quantity of viable bacteria peaked in 24 hr when only *Lactobacillus* was inoculated in the rice acid fermentation. Rice acid fermented with four grains showed the consistent growth curves when *L. paracasei* H4‐11 was inoculated in rice acid. However, the quantities of colonies were different, indicating that *L. paracasei* H4‐11 had the different growth mechanism among different raw materials. Rice acid, respectively, inoculated with *L. fermentum* D1‐1 and *L. paracasei* H4‐11 showed significant differences in the quantities of LAB, indicating that the fermentation mechanisms of the strains were different. Interestingly, *L. fermentum* D1‐1 was more suitable for the fermentation of barley rice and brown rice. The colonies in rice acid fermented with barley rice and brown rice at 24 hr were significantly more than those in rice acid fermented with other grains, but rice acid fermented with four cereals showed the consistent trend of the quantity of colonies after 96‐hr fermentation. In rice acid fermented with *L. paracasei* H4‐11 (or *L. fermentum* D1‐1) and *K. marxianus* L1‐1, the quantities of LAB and yeasts showed the similar changing trends. In the first day, the quantities of LAB and yeasts showed an upward trend since LAB strains and yeast strains could supplied nutrients to each other. In the second day, the quantities of LAB and yeasts declined because the two strains competed substrates. After three days, an upward trend appeared again since the accumulation of various nutrients in the fermentation system provided a suitable growth environment for LAB and yeasts. However, after 4 days, LAB and yeasts declined since the nutrients generated by yeasts were mainly utilized by LAB (Mugula et al., 2003). Interestingly, no growth promotion phenomenon was observed in the second and third days. The symbiosis and competition phenomenon was observed in the early fermentation stage. Ishii et al. (1999) had discovered that *L. paracasei subsp. tolerans* and *K. marxianus* promoted the symbiosis with each other during the fermentation of Chigo. Until the fourth day of the late fermentation period, yeasts appeared to promote the growth of LAB. Therefore, the taste and aroma of rice acid showed a more stable flavor after four days, indicating that 4 days could be the proper end of rice acid fermentation.

#### 3.8.2 L‐lactic acid‐producing performance characteristics in rice acid fermented with different raw materials

**FIGURE 7:**
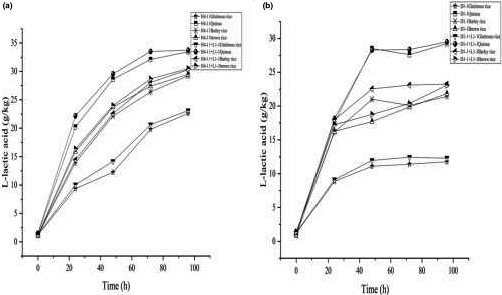
(a) The capacity of L‐lactic acid production of *L. paracasei* H4‐11 and *K. marxianus* L1‐1 fermented rice acid by different raw materials included glutinous rice, quinoa, barley rice, and brown rice. (b) The capacity of L‐lactic acid production of *L. fermentum* D1‐1 and *K. marxianus* L1‐1 fermented rice acid by different raw materials included glutinous rice, quinoa, barley rice, and brown rice

*L. paracasei* H4‐11 and *L. fermentum* D1‐1 showed different L‐lactic acid production properties in the fermentation with different raw materials: glutinous rice, quinoa, barley rice, and brown rice (Figure 7). *Lactobacillu*s had been reported to produce L‐lactic acid (Bernardo et al., 2016). The purity of L‐lactic acid was 96% in our study. Quinoa had the highest L‐lactic acid production performance. When the substrate had a low content of sugar, the concentrations of L‐lactic acid in rice acid, respectively, fermented with *L. paracasei* H4‐11 and *L. fermentum* D1‐1 reached 33.44 g/kg and 29.22 g/kg. Notably, the concentration of L‐lactic acid produced by LAB and yeasts in the mixed fermentation was greater than the result of Freire et al. (2017). Meanwhile, the concentration of L‐lactic acid produced by *L. paracasei* H4‐11 was greater than that of L‐lactic acid produced by *L. fermentum* D1‐1 in our study. *L. paracasei* produced L‐lactic acid through facultative heterolactic fermentation. L‐lactic acid in the fermentation systems of H4‐11 and D1‐1 increased to different degrees since *K. marxianus* L1‐1 could decompose grain glucose into L‐lactic acid through a series of physiological and biochemical reactions. The synergistic fermentation of *L. paracasei* H4‐11 and *K. marxianus* L1‐1 could increase the production of L‐lactic acid. Consistently, the previous study (Plessas et al., 2008) demonstrated that the high lactic acid content was achieved when *K. marxianus* was used together with *L. delbrueckii ssp. Bulgaricus*. Due to the different fermentation system used in this study, the acid yield was not comparable to the previous study. However, it had been confirmed that *K. marxianus* L1‐1 combined with *L. paracasei* H4‐11 (and *L. fermentum* D1‐1) could promote L‐lactic acid production. Interestingly, the concentrations of L‐lactic acid in quinoa and barley rice acid fermented with *L. fermentum* D1‐1 in 72 hr showed a downward trend. This phenomenon might be related to the large‐scale growth of LAB in the lactic acid fermentation system in this fermentation stage. The LAB decomposed glucose after 96 hr of fermentation, and the concentrations of L‐lactic acid in quinoa and glutinous rice rice acid, respectively, reached 33.73 g/kg and 23.09 g/kg. It could be inferred that after the fermentation process was nearly completed and the nutrients had been consumed by saccharification, alcoholization, and acidification, the maximum acid production was realized.

#### 3.8.3 Antioxidant capacity of DPPH free radical scavenging ability in rice acid fermented with different raw materials

**FIGURE 8:**
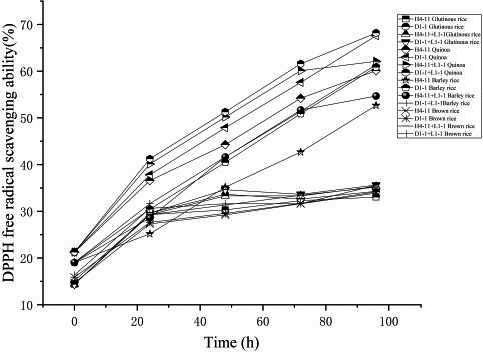
The DPPH free radical scavenging ability in different strains fermented rice acid (*L. paracasei* H4‐11, *L. fermentum* D1‐1, *L. paracasei* H4‐11, and *K. marxianus* L1‐1, *L. fermentum* D1‐1, and *K. marxianus* L1‐1) by 4 raw materials included glutinous rice, quinoa, barley rice, and brown rice

*L. paracasei* H4‐11 and *L. fermentum* D1‐1 strains had a certain difference in DPPH free radical scavenging ability (Figure 8), and their DPPH free radical scavenging ability was the highest in quinoa rice acid. In the fermentation with *L. fermentum* D1‐1, the DPPH free radical scavenging ability reached 67.57% after 96 hr. In the rice acid fermentation inoculated with *L. paracasei* H4‐11, the DPPH free radical scavenging ability reached 60.1%, whereas DPPH free radical scavenging ability in rice acid fermented with glutinous rice, and *L. paracasei* H4‐11 and *L. fermentum* D1‐1 were, respectively, 34.27% and 33.05%. The two strains showed the significant difference in the DPPH free radical scavenging ability (*p* < .05). Among the four rice acid raw materials, glutinous rice and brown rice realized poor DPPH free radical scavenging ability in rice acid since the differences in the raw materials of brown rice and glutinous rice led to inconsistent antioxidant properties. Meanwhile, the DPPH free radical scavenging capacity realized the significant difference among different strains and continuously increased. In rice acid synergistically fermented with LAB and yeasts, glutinous rice and brown rice showed the close antioxidant capacity, whereas quinoa and barley rice realized the better antioxidant activity. It was worth mentioning that the free radical scavenging ability in rice acid synergistically fermented with *L. fermentum* D1‐1 and *K. marxianus* L1‐1 strains showed the better antioxidant properties than that in rice acid synergistically fermented with *L. paracasei* H4‐11 and *K. marxianus* L1‐1. The protein hydrolysates of *K. marxianus* L1‐1 and *L. fermentum* D1‐1 showed the higher combined antioxidant ability, and DPPH free radical scavenging capacity in rice acid of quinoa and glutinous rice, respectively, reached 68.18% and 35.63%. This was consistent with the evaluation results of antioxidant and ACE‐inhibitory activity of protein hydrolysates of *K. marxianus* in the previous study (2018). However, the interpretation of this result needs further study.

#### 3.8.4 Electronic tongue measurement in rice acid fermented with different raw materials

**FIGURE 9:**
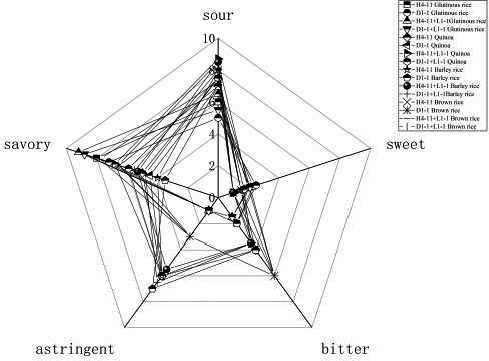
The analysis of taste substances in different strains fermented rice acid (*L. paracasei* H4‐11, *L. fermentum* D1‐1, *L. paracasei* H4‐11 and *K. marxianus* L1‐1, *L. fermentum* D1‐1, and *K. marxianus* L1‐1) by 4 raw materials included glutinous rice, quinoa, barley rice, and brown rice

The sweetness intensity showed no significant difference among rice acid fermented with different raw materials (Figure 9), indicating that the rice acid fermentation system was mature so that there was no excess starch to be converted into glucose. The most important flavor was sour, which showed significant differences among various samples. Among rice acid fermented with *L. fermentum* D1‐1, the sourness intensity of barley rice and quinoa was more prominent than that of glutinous rice and brown rice inoculated with *L. paracasei* H4‐11. The sourness intensity in rice acid synergistically fermented with *L. paracasei* H4‐11, and *K. marxianus* L1‐1 was more prominent than that in rice acid inoculated with *L. paracasei* H4‐11, indicating that *L. paracasei* H4‐11 and *K. marxianus* L1‐1 played a crucial role in the sourness formation in the rice acid fermentation. The sourness intensity measured by electronic tongue was consistent with the determined concentration of L‐lactic acid in this study. Interestingly, in terms of the savory intensity, the flavor in rice acid synergistically fermented with LAB and yeasts was stronger than that in rice acid fermented with single LAB, indicating that *K. marxianus* L1‐1 could improve the flavor of rice acid. Consistently, a recent study (Martínez et al., 2017) demonstrated that *K. marxianus* produced aroma compounds such as mellow and ester could promote the flavor accumulation. The previous study (Ballesteros, Oliva, Negro, Manzanares, & Ballesteros, 2004) proved that *K. marxianus* could produce ethanol during saccharification and fermentation in lignocellulosic materials. The bitterness intensity and astringent taste intensity showed the significant differences between rice acid of quinoa and barley rice mainly due to the taste of the raw materials themselves. Both quinoa and barley rice had a high astringent taste intensity. Glutinous rice exhibited a unique flavor characteristic, indicating that glutinous rice was more suitable for the joint fermentation of *Lactobacillus* and *K. marxianus* L1‐1. The different intensities of sourness, sweetness, bitterness, astringent taste, and savory indicated that glutinous rice was more suitable for the fermentation system of *L. paracase* H4‐11 and *K. marxianus* L1‐1. The flavors produced by the single inoculation with *L. paracasei* or *K. marxianus* had been reported (Crafack et al., 2013). However, the mixed fermentation flavors of *L. paracasei* and *K. marxianus* were not reported. Therefore, the collaborative fermentation of *L. paracase* H4‐11 and *K. marxianus* L1‐1 selected in this study provides a theoretical support for the unique flavors of Chinese traditional fermented rice acid.

## 4 CONCLUSION

In this study, we screened L‐lactic acid‐producing lactic acid bacteria and aroma‐producing yeasts from rice acid and applied them improving the quality of rice acid for the first time. According to conventional morphological, physiological, and biochemical characteristics, L‐lactic acid production capacity, antioxidant capacity, and flavor characteristics, 16S rRNA, and fungal ITS sequence analysis was performed to screen the strains with high L‐lactic acid production performance. Four LAB strains were, respectively, identified as *L. paracasei* H4‐11, *L. fermentum* D1‐1, *L. casei* H1‐8, and *L. reuteri* H2‐12, and a yeast strain was identified as *K. marxianus* L1‐1. Glutinous rice, quinoa, barley rice, and brown rice were selected to perform fermentation experiments. Rice acid synergistically fermented with *L. paracasei* H4‐11 and *K. marxianus* L1‐1 in glutinous rice showed the highest L‐lactic acid concentration (23.09 g/kg). Rice acid fermented with both *L. paracasei* H4‐11 and *K. marxianus* L1‐1 in glutinous rice showed a unique flavor and high antioxidant capacity. Therefore, *L. paracasei* H4‐11 and *K. marxianus* L1‐1 were potential strains for the fermentation of rice acid. We will further explore the mechanism of synergistic fermentation of *L. paracasei* H4‐11 and *K. marxianus* L1‐1 in the future study.

## CONFLICTS OF INTEREST

All authors declare that they have no conflicts of interest.

## ETHICAL STATEMENTS

This study does not involve any human or animal testing.
